# Hemiarch Versus Arch Replacement in Acute Type A Aortic Dissection: Is the Occam’s Razor Principle Applicable?

**DOI:** 10.3390/jcm11010114

**Published:** 2021-12-26

**Authors:** Igor Vendramin, Daniela Piani, Andrea Lechiancole, Sandro Sponga, Concetta Di Nora, Francesco Londero, Daniele Muser, Francesco Onorati, Uberto Bortolotti, Ugolino Livi

**Affiliations:** 1 Division of Cardiac Surgery, Cardiothoracic Department, University Hospital of Udine, 33100 Udine, Italy; daniela.piani@asufc.sanita.fvg.it (D.P.); andrea.lechiancole@asufc.sanita.fvg.it (A.L.); sandro.sponga@asufc.sanita.fvg.it (S.S.); concetta.dinora@asufc.sanita.fvg.it (C.D.N.); francesco.londero@asufc.sanita.fvg.it (F.L.); uberto48@gmail.com (U.B.); ugo.livi@asufc.sanita.fvg.it (U.L.); 2 Division of Cardiology, Cardiothoracic Department, University Hospital of Udine, 33100 Udine, Italy; daniele.muser@asufc.sanita.fvg.it; 3 Division of Cardiac Surgery, Azienda Ospedaliero-Universitaria di Verona, 37100 Verona, Italy; francesco.onorati@univr.it; 4 Division of Cardiac Surgery, Department of Medical Area (DAME), University of Udine, 33100 Udine, Italy

**Keywords:** acute aortic dissection, hemiarch replacement, aortic arch replacement

## Abstract

Background and aim of the study: In patients with acute Type A aortic dissection (A-AAD) whether repair should be limited to ascending aorta/hemiarch replacement or extended to include the aortic arch is still debated. We have analyzed our experience to compare outcomes of patients with A-AAD treated with these 2 different surgical strategies. Methods: From 2006 to 2020, a total of 213 patients have undergone repair of A-AAD at our Center; in 163 of them ascending aorta/hemiarch replacement (Group 1) and in 75 ascending aorta and arch replacement (Group 2) were performed. The primary endpoint was early survival and secondary endpoints late survival, freedom from late complications and reoperations. Patients were compared according to era of operation: 2006 to 2013 (Era 1) and 2014 to 2020 (Era 2). Results: Overall hospital mortality was 12% and 5% in Group 1 and 2; mortality remained stable in Era 1 and 2 for Group 1 (15%), while it decreased from 8% to 1% in Group 2 patients (*p* = 0.24). Actuarial survival at 5 and 10 years is 72 ± 4% and 49 ± 5% in Group 1 and 77 ± 6% and 66 ± 9% in Group 2 (*p* = 0.073). Actuarial freedom from reoperation in the entire series is 94 ± 2% and 92 ± 3% at 5 and 10 years. Freedom from reoperation at 5 and 10 years is 92 ± 2% and 89 ± 3% in Group 1 and 98 ± 1% at all intervals in Group 2 (*p* = 0.068). Conclusions: An aggressive approach to A-AAD provides superior long-term results without increasing mortality. Furthermore, arch replacement during A-AAD repair represents a more stable solution with lower incidence of late aortic-related complications. Immediate aortic arch replacement should be considered in the treatment of A-AAD especially in experienced centers.

## 1. Introduction

Acute type A aortic dissection (A-AAD) is a real surgical emergency with the primary objective to obtain patient survival. After the first attempts to treat A-AAD, initially by aortic fenestration and then by ascending aorta resection and graft interposition, as reported by DeBakey et al. [1,2,3], surgical techniques have evolved considerably in the following decades. Isolated replacement of the ascending aorta, at times including the hemiarch, is still a valid option supporting the strategy for a low risk, straightforward operation in many patients with A-AAD [4]. Significant reduction of hospital mortality achieved in most recent years, has stimulated many centers to perform routine total aortic arch replacement at initial repair in patients presenting with A-AAD, even if the arch is not severely dilated or is not involved by intimal tears. Supporters of this more aggressive approach concur on the positive effects represented by a better long-term survival and lower need for reoperation due to reduction of postoperative aortic-related complications [5,6].

The real advantage of a limited versus a more challenging initial approach in the treatment of A-AAD is still an open question since substantial and conclusive data in favor of either strategy are not yet available.

To provide further data to help clarify this still unsolved debate, we have reviewed our experience comparing two series of patients with A-AAD undergoing ascending aorta associated to either hemiarch or total arch replacement analyzing patient early and long-term outcomes.

## 2. Material and Methods

We have considered all patients referred to our Center with A-AAD who have been operated from 2006 to 2020. A total of 213 patients were analyzed, 163 of whom had ascending aorta and hemiarch replacement (Group 1) and 75 in whom the ascending aorta and arch were simultaneously replaced at initial repair. The 2 Groups were compared considering as primary endpoint early mortality and morbidity and as secondary endpoints late survival and freedom from late complications or aortic reoperations. Patients were also divided and compared according to the era of operation, namely those treated between 2006 and 2013 (Era 1) and those from 2014 to 2020 (Era 2).

The local Ethical Committee gave consent for this study waiving the need for patient consent due to its retrospective nature.

*Patient characteristics*: Group 1 patients were older with a median age of 69 years (range 43 to 89 years) compared to those of Group 2 (median age of 58 years, range 28 to 78 years) (*p* < 0.01); 106 Group 1 patients were males (65%) vs. 55 (77%) of Group 2. Main preoperative data, including clinical presentation, neurological status and risk factors are summarized in Table 1.

*Surgical strategy*: All patients presenting with A-AAD were operated on an emergency basis as soon as the diagnosis was confirmed by angio-computed tomography (CT). In those with hemodynamic instability the suspect of A-AAD relied only on typical symptoms and clinical examination, the diagnosis being confirmed by preoperative 2D transthoracic or intraoperative trans-esophageal echo. Upon arrival, in all patients, evaluation of the neurological status was performed to evidence signs of cerebral malperfusion.

Two operative strategies were employed mainly dictated by preoperative imaging, intraoperative findings and surgeon’s experience. Initially, most patients underwent ascending aorta replacement, in all cases extended to the hemiarch, especially if the entrance tear was located in the ascending aorta and in the absence of dilatation of the aortic arch. When multiple tears were present or A-AAD started in the arch both the ascending aorta and arch were replaced.

Surgical techniques have been described in part elsewhere [7]. All operations were performed through a median sternotomy on cardiopulmonary bypass (CPB) instituted by cannulating the femoral vessels while in recent years the right axillary artery was routinely utilized. Deep hypothermic circulatory arrest and retrograde cerebral perfusion were used initially and then substituted by moderate hypothermia and continuous antegrade selective cerebral perfusion through the right axillary artery and direct cannulation of the left carotid artery. After preparing the distal aortic stump with biological glue and reinforcing it with strips of Teflon, the aortic arch was replaced mainly with the classic elephant trunk (ET) procedure and more recently with the frozen ET (FET) using a quadrifurcated graft. During the distal suture a tip-cut Foley catheter was inflated into the graft for splanchnic perfusion which was then replaced by cannulating the lateral branch of the graft. All other anastomoses and surgical procedures on the aortic root or valve were performed during rewarming.

Patients having aortic valve or root replacement with a mechanical prosthesis were routinely kept on life-long oral anticoagulants; in all the others antiplatelet medications were administered unless specific thrombotic risk factors were present [8].

*Patient follow-up*: Patients were re-evaluated clinically and by transthoracic 2D echo after one and six months and yearly thereafter. Since 2010, CTs were performed annually for the first 5 years and then whenever needed. Medical records were reviewed to assess incidence and causes of any postoperative complication. Causes of death were confirmed from medical records, death certificates, post-mortem reports and contact with family members and referring physicians.

*Statistical analysis*: Continuous variables, expressed as means ± standard deviations if normally distributed or medians (minimum–maximum range) if not, were tested for normal distribution using the 1-sample Kolmogorov–Smirnov test. Continuous variables were compared using independent-sample parametric (unpaired Student t) or non-parametric (Mann-Whitney U) tests. Categorical data, expressed as counts and percentages, were compared using Chi-Square or Fisher Exact test when appropriate. Univariable and multivariable logistic regression analysis was performed to evaluate the association between baseline covariates and 30-days mortality. All potential confounders were initially entered into the multivariable model on the basis of known clinical relevance; then a model reduction was performed by excluding variables with a *p*-value > 0.20 based on the log-likelihood test. Survival curves were generated by Kaplan–Meier method and compared by log rank test. As part of a sensitivity analysis the univariable model was also adjusted using inverse-probability weighting by a propensity score, taking into account 13 baseline covariates including age, gender, obesity, smoking habit, arterial hypertension, dyslipidemia, diabetes mellitus, chronic kidney disease, atrial fibrillation, connective tissue disorder, use of preoperative oral anticoagulation, chronic obstructive pulmonary disease and previous cardiac surgery. A competing risk analysis, based on the Fine and Gray risk time-to-event model, was used for analysis of time to reintervention while taking the competing risk of death into account [9]. Comparison between curves was made by the Gray’s test [10], while event times were measured from the date of surgery. Two-tailed tests were considered statistically significant at 0.05 level. Statistical analysis was performed using IBM-SPSS software Version 22.0 (IBM Corp., Armonk, NY, USA) and in R version 4.0.3 software (R Foundation for Statistical Computing, Vienna, Austria).

## 3. Results

Surgical data: Main intra and postoperative data are shown in Table 2. Cannulation of the right axillary artery for CPB was used more frequently in Group 2 (88%) than in Group 1 patients (51%) (*p* < 0.001) while selective antegrade cerebral perfusion was employed in all patients with arch and in 82% of those with hemiarch replacement (*p* < 0.001). In 36 of Group 2 patients (48%) a classic ET procedure was performed while in 15 (47%) the arch was replaced with a FET. Patients of Group 2 had significantly higher CPB, aortic cross-clamp and circulatory arrest times (*p* < 0.001) when compared to Group 1 patients.

Surgical results according to the era of operation (2006–2013 vs. 2019–2010) are indicated in Table 3. Both in Era 1 and 2 there was an evident increase in the use of axillary artery cannulation and of selective antegrade cerebral perfusion in Group 2 compared to Group 1 patients. CPB and aortic cross-clamp times were significantly reduced in patients having either hemiarch or arch replacement from era 1 to era 2. Arch replacement was mainly performed during era 2 (55 patients, 42%) than in era 1 (20 patients, 19%) (*p* < 0.001).

All patients having arch replacement in Era 2 received a FET which generally was preferred to the classic ET procedure. Main clinical and surgical characteristics of patients receiving a FET procedure are shown in Table 4.

*Early and late results*: Overall hospital (30-day) mortality was 12% and 5% in Group 1 and 2, respectively (*p* = 0.16). Nineteen patients of Group 1 died because of aortic rupture (7 patients), neurologic complications (3 patients), heart failure (4 patients), multi-organ failure (4 patients) and mesenteric ischemia (1 patient); 4 patients of Group 2 died because of aortic rupture (1 patient), neurologic complications (1 patient) and 2 patients died in the early postoperative hours.

Incidence of major postoperative complications was similar in the 2 Groups being mainly represented by acute renal insufficiency (42% vs. 40%) and by permanent neurologic deficit (10% vs. 14%). Median hospital stay was significantly superior for Group 2 patients (17 vs. 22, *p* < 0.01). Overall 150 (63%) experienced postoperative complications without a significant difference between the two groups: 98 (60%) patients in the hemiarch group and 52 (69%) in the arch replacement group (*p* = 0.17).

Overall hospital mortality improved from 12% in Group 1 to 5% in Group 2 (*p* = 0.16). Hospital mortality remained stable in era 1 and 2 for Group 1 (15%), while it decreased from 8% to 1% in Group 2 patients (*p* = 0.24). Median hospital stay was superior for Group 2 patients compared to those of Group 1 both in Era 1 and 2.

Mean follow-up was 5 ± 4 in Group 1 and 4 ± 3 years in Group 2 patients (*p* = 0.019). There were 59 late deaths, 48 in Group 1 and 11 in Group 2. Main causes of death were sudden death in 11, cerebral damage in 4 and heart failure in 2; in 10 patients the cause of death is unknown while in 32 it was not aortic or cardiac-related.

**Figure 1:**
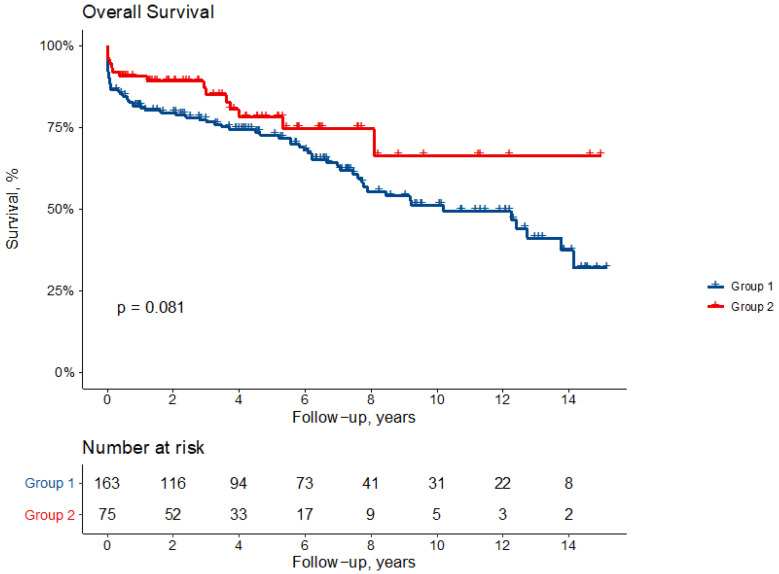
Actuarial survival following repair of type A acute aortic dissection according. to the type of initial repair: ascending hemiarch replacement (Group 1) or arch replacement (Group 2).

Actuarial survival at 1, 5 and 10 years is 81 ± 3%, 72 ± 4% and 49 ± 5% in Group 1 and 90 ± 3%, 77 ± 6% and 66 ± 9% in Group 2 patients (*p* = 0.073) (Figure 1).

After multivariable adjustment tamponade/shock at presentation (OR 7.30, 95% CI 2.42–22.00; *p* < 0.001) and ACC time (OR 1.04, 95%CI 1.02–1.06; *p* < 0.001) were independent predictors of 30-day mortality while aortic arch replacement was associated with a 92% lower 30-day mortality compared to hemiarch replacement (OR 0.08, 95% CI 0.01–0.63; *p* = 0.02) (Table 5). Inverse probability weighting analysis confirmed a significant association of arch replacement with lower mortality (OR 0.33, 95% CI 0.16–0.70, *p* = 0.004).

**Figure 2:**
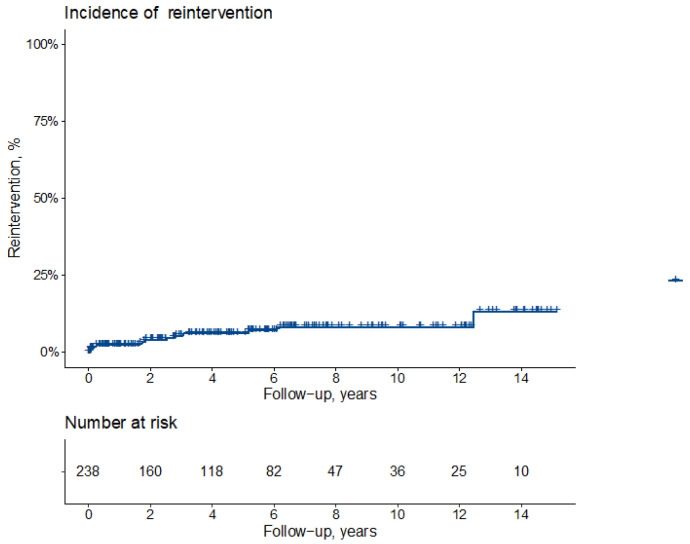
Cumulative incidence of reintervention (all-cause death as competing risk).

**Figure 3:**
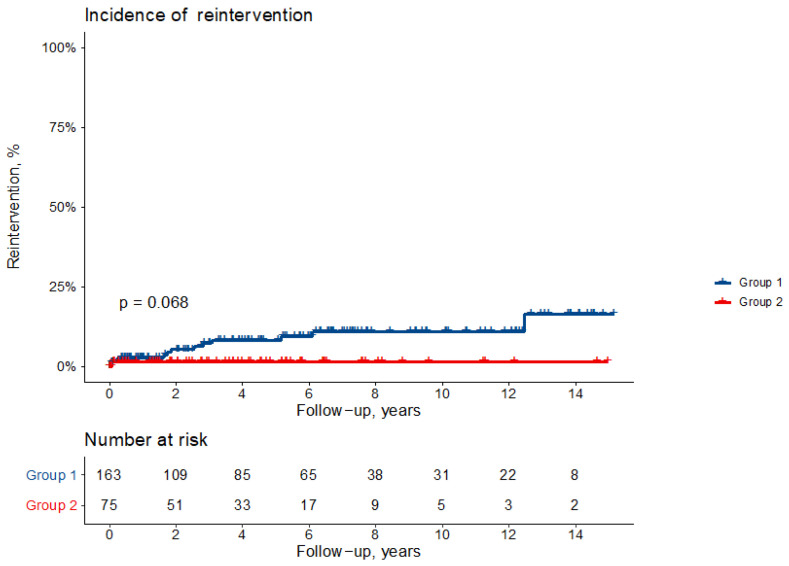
Cumulative incidence of reintervention (all-cause death as competing risk) according to the study group: hemiarch replacement (Group 1) or arch replacement (Group 2).

Actuarial freedom from reoperation in the entire series is 97 ± 1%, 94 ± 2% and 92 ± 3% at 1, 5 and 10 years (Figure 2). Freedom from reoperation at 1, 5 and 10 years is 97 ± 2%, 92% ± 2% and 89 ± 3% in Group 1 and 98 ± 1% at all intervals (*p* = 0.068) (Figure 3).

Number and type of reoperation in each group is shown in Table 6.

## 4. Discussion

In patients with A-AAD immediate surgical treatment is mandatory, as soon as the correct diagnosis is obtained, in order to prevent death mainly from aortic rupture and cardiac tamponade. In fact, A-AAD, when left untreated, shows a mortality rate of 1–2% per hour within the first 24 h, and a mortality rate of up to 50–74% during the acute phase [11]. Surgery is therefore mainly a life-saving procedure and for such reason limited replacement of the ascending aorta has represented in the past the standard strategy in patients with A-AAD. Increasing experience, with improvements in diagnosis, intraoperative and postoperative management, has led to better early and late outcomes; in particular, advanced techniques in myocardial, cerebral and splanchnic protection have stimulated surgeons to attempt more complex repairs such as those not limited to reconstruction of the ascending aorta but also extended to total arch replacement [5,12].

In many patients with A-AAD it is still reasonable to limit repair to replacement of the ascending aorta with possible hemiarch extension [4]; however, when the aortic arch is significantly dilated, is the site of entry intimal tears and dissection of the epiaortic vessels occurs with cerebral malperfusion, arch replacement appears generally unavoidable [5].

Supporters of immediate aortic arch replacement for A-AAD repair are justified by considering that A-AAD survivors are at risk of late reoperations due to possible expansion of the distal false lumen and pseudoaneurysm formation [5,13], and that persistence of residual false lumen has been reported to be an independent risk factor for poor long-term survival [14,15,16,17,18]. There is current evidence that the rate of reoperations is higher in patients with limited supra-commissural replacement of the ascending aorta compared to those who had arch replacement at initial repair; in addition, late distal reoperation on the arch often represents a cumbersome and challenging procedure [18,19]. It must however also be recognized that in many patients, presenting with A-AAD, the decision on the best surgical option must be taken in an emergency situation and at times by less experienced surgeons. For all the above mentioned reasons the optimal surgical strategy in patients with A-AAD is still a matter of debate [4,5,6].

In the present report we have compared patients having repair of A-AAD either by graft replacement of the ascending aorta and hemiarch or ascending aorta and arch, analyzing a single center experience. Furthermore, patients were evaluated also according to two different time intervals, to verify whether changes in patient selection, surgical strategies and technical modifications might have resulted in different outcomes. From our analysis it appears that through the years there have been important technical modifications. These are represented by a more extended and significant use of the right axillary artery for arterial cannulation for CPB, with progressive reduction of peripheral cannulation through a femoral artery. Axillary artery cannulation has been used in most cases of patients having arch replacement (Group 2), but a significant trend has been observed also in those of Group 1. Moreover, it also appears that selective antegrade cerebral perfusion, coupled with splancnic protection and immediate systemic reperfusion, upon completion of the distal aortic suture line, has become and is currently our procedure of choice for neurologic and systemic organ protection, as favored also in many other centers [20,21,22].

Aortic arch surgery has rapidly evolved from the technical point of view in recent years. The technique of an island of arch containing the brachiocephalic vessels reimplanted on the replacement graft [23] has been outdated by the separate reattachment of the arch vessel using a tri- or quadrifurcated graft [24]. Methods to stabilize the distal false lumen utilizing metallic stents have been used with initial promising results but did not stand the test of time with many postoperative device-related complications reported [25,26]. In arch surgery the original description by Borst et al. of the ET technique in 1983, has provided the means for effective arch replacement preparing a distal landing zone for possible future reoperations on the thoracic aorta [27]. Maintaining the ET principle, the FET has been introduced in the surgical armamentarium which, using a hybrid graft, offers a more rigid distal support for possible late, low risk endovascular procedures [28]. The use of frozen ET has provided excellent results in the treatment of A-AAD with a reported operative mortality <7%, 74% survival and 84% freedom from reoperation at 8 years in some series [29]. It must be underlined that also in our experience the classic ET has been abandoned in favor of the FET which is currently routinely used in all patients requiring aortic arch replacement; it has been demonstrated that the frozen ET can indeed be used also in complex settings with appealing results [30,31].

With increasing experience our surgical approach to patients with A-AAD has changed over time. In the first period (Era 1) most patients had repair limited to graft replacement of the ascending aorta; however, it is noteworthy that, even in this setting, repair was extended in all patients to the hemiarch to achieve a more complete aortic replacement. Subsequently, the number of total arch replacements have increased and indeed most of such procedures have been performed in the most recent years (Era 2).

Total arch replacement is certainly a complex procedure but our experience demonstrates that the results obtained can be quite gratifying even in emergency settings and when dealing with complex scenarios as those frequently found intraoperatively in A-AAD. Overall 30-day mortality was 12% in Group 1 and 5% in Group 2. Considering the two different time periods, mortality has decreased in both Groups, regardless of the surgical procedure, being lower in Group 2 compared to Group 1 in the most recent era (1% vs. 6%). The apparent paradox of a lower mortality for a more complex operation may be probably explained by the fact that Group 1 patients were significantly older than those of Group 2. However, we consider as another determinant of such results also the changes which have occurred in our organization in the recent years with creation of a regional network to assure a fast diagnosis and referral. Furthermore, in our center an aortic team has been organized which includes all staff members, adequately trained, vascular surgeons, cardiologists, anesthesiologists and interventional radiologists with established protocols to provide to each patient a tailored surgical strategy.

This study indicates that also late survival is superior when an extended procedure is performed to repair A-AAD. In fact, at 10 years the observed actuarial survival in Group 1 was 49 ± 5% while it was 66 ± 9% in Group 2, with a similar mean follow-up interval. This indicates that a more radical approach provides definite benefits in patients with A-AAD without increasing the operative risk; furthermore, the risk of possible future reoperations due to aortic-related complications, such as progression of dissection, distal dilatation or pseudoaneurysm formation, appears minimized.

The major limitation of the present paper may be recognized in its retrospective nature; considering the peculiarity of A-AAD, due to its possible different clinical presentations and the heterogenicity of the underlying pathological substrates, it is evident that a prospective randomized study may be impossible to perform, especially should the superiority of an immediate radical approach be demonstrated in the treatment of A-AAD. Furthermore, to obtain more meaningful data we have not considered patients operated at the very beginning of our experience which dates back to 1977; this allowed to obtain 2 groups of patients with more comparable characteristics and more evenly distributed through different time intervals.

In conclusion, we have reviewed a series of patients treated for A-AAD during over 2 decades comparing those having limited ascending aorta and hemiarch with those having total arch replacement at index operation. Our results, which support an aggressive approach to A-AAD with superior long-term outcomes, are most likely favored by the peculiar organization established in our center for the care of A-AAD patients. Furthermore, arch replacement during A-AAD repair provides a more stable solution with lower incidence of late aortic-related complications which can be even treated with endovascular procedures avoiding the need for complex and hazardous surgical reoperations. We believe that aortic arch replacement should be strongly considered especially in experienced centers as initial procedure in the treatment of A-AAD.

## 5. Addendum

It is generally attributed, probably erroneously, to William of Occam the statement ‘*Entia non sunt multiplicanda, praeter necessitatem*’ (Entities should not be multiplied without necessity) [32], also better known as Occam’s razor rule *‘the simplest solutions are always the better’.* The results of the present analysis demonstrate that this principle is not always applicable to the treatment of A-AAD.

## Figures and Tables

**Table 1:** Main preoperative patient data.

	Group 1 (*n* = 163)	Group 2 (*n* = 75)	*p* Value
Median age, years (min–max)	69 (43–89)	58 (28–78)	<0.001
Male sex, *n*. (%)	106 (65)	57 (77)	0.057
**Risk factors**			
Dyslipidemia, *n*. (%)	28 (17)	10 (14)	0.49
Obesity, *n*. (%)	32 (20)	20 (28)	0.19
Diabetes, *n*. (%)	11 (7)	2 (3)	0.21
Hypertension, *n*. (%)	124 (77)	58 (80)	0.62
Chronic kidney damage, *n*. (%)	7 (4)	5 (7)	0.42
Chronic AF, *n*. (%)	20 (12)	6 (8)	0.35
Smoking habitus, *n*. (%)	52 (32)	23 (32)	0.93
Chronic OAC, *n* (%)	19 (12)	7 (10)	0.63
BAV, *n*. (%)	11 (7)	1 (1)	0.11
Connective tissue disorder, *n*. (%)	2 (1)	2 (3)	0.41
Previous cardiac surgery, *n*. (%)	8 (5)	3 (4)	1
**Clinical presentation**			
Syncope, *n*. (%)	30 (19)	12 (17)	0.74
Transient neurological deficit, *n*. (%)	28 (18)	19 (27)	0.11
Coma, *n*. (%)	1 (1)	3 (4)	0.09
Cardiac tamponade/shock, *n*. (%)	61 (38)	20 (28)	0.15
Chest pain, *n*. (%)	125 (79)	59 (84)	0.36
AR ≥ moderate, *n*. (%)	41 (26)	22 (34)	0.23
Acute kidney failure, *n*. (%)	16 (14)	12 (19)	0.45

AF = Atrial fibrillation; OAC = Oral anticoagulants; BAV = Bicuspid aortic valve; AR = Aortic regurgitation.

**Table 2:** Summary of surgical data.

	Group 1 (*n* = 163)	Group 2 (*n* = 75)	*p* Value
**Arterial cannulation**			<0.001
Right axillary, *n*. (%)	83 (51)	65 (88)	
Femoral, *n* (%)	73 (44)	8 (11)	
Other, *n*. (%)	7 (4)	1 (1)	
**Cerebral perfusion**			<0.001
Retrograde	29 (18)	0	
Selective antegrade	132 (82)	75 (100)	
**Associated procedures**			
AVR	9 (1 Djumbodis) (6)	1 (1)	0.14
Bentall	18 (5 CABG) (11)	3 (1 CABG) (4)	0.08
Tirone-David	2 (1 Djumbodis) (1)	5 (7)	0.002
Yacoub	2 (1)	-	-
CABG	5 (3)	-	-
**Arch surgery**			
Classic ET, *n*. (%)	-	36 (48)	
Frozen ET, *n*. (%)	-	15 (20)	
TAR, *n*. (%)		24 (32)	
**Intraoperative data**			
Median CPB time, minutes (min–max)	185 (102–444)	232 (152–612)	<0.001
Median ACC time, minutes (min–max)	88 (40–330)	148 (65–340)	<0.001
Median arrest time, minutes (min–max)	38 (5–90)	48 (13–228)	<0.001
Median core temperature, °C (min–max)	25 (20–31)	25 (22–28)	0.51
**Postoperative complications**			
Chest re-exploration, *n*. (%)	32 (20)	8 (11)	0.11
Splanchnic ischemia, *n*. (%)	6 (4)	3 (4)	1
Atrial fibrillation, *n*. (%)	63 (40)	30 (43)	0.67
Acute kidney injury, *n*. (%)	65 (42)	42 (40)	0.013
Dialysis, *n*. (%)	26 (17)	18 (26)	0.10
Permanent neurologic deficit, *n*. (%)	16 (10)	10 (14)	0.36
**Postoperative course**			
Median ICU stay, days (min–max)	5 (1–129)	7 (2–57)	0.005
Median hospital stay, days (min–max)	17 (1–129)	22 (10–61)	<0.001
30-day mortality, *n*. (%)	19 (12)	4 (5)	0.16

AVR = Aortic Valve Replacement; CABG = Coronary Artery Bypass Graft; ET = Elephant trunk; CPB = Cardiopulmonary; ACC = Aortic cross-clamp; ICU = Intensive care unit.

**Table 3:** Summary of operative data according to the surgical era.

	Era 1 (2006–2013)			Era 2 (2014–2020)		
	AA + Hemiarch *n* = 87	AA + Arch *n* = 20	*p*	AA + Hemiarch *n* = 76	AA + Arch *n* = 55	*p*
**Intraoperative data**						
**Arterial cannulation**			<0.001		52 (95) 3 (5)	0.13
Right axillary, *n*. (%)	21 (24)	14 (70)		62 (81)		
Femoral, *n* (%)	64 (74)	5 (25)		9 (12)		
Other, *n*. (%)	2 (2)	1 (5)		5 (7)		
**Cerebral perfusion**					56 (100)	
Retrograde				75 (99)		1
Selective antegrade				1 (1)		-
**Arch surgery**	-	13 (65)	-	-		
Classic ET, *n*. (%)					23 (42)	-
Frozen ET, *n*. (%)					15 (27)	-
Median CPB time, minutes (min–max)	210 (128–444)	319 (191–428)	<0.001	160 (108–373)	225 (170–612)	<0.001
Median ACC time, minutes (min–max)	88 (40–330)	188 (65–306)	0.001	93 (45–220)	149 (83–340)	<0.001
Median arrest time, minutes (min–max)	42 (5–90)	65 (13–228)	<0.001	36 (17–84)	48 (19–130)	0.04
Median core temperature, °C (min–max)	24 (20–26)	25 (22–25)	0.74	26 (24–31)	26 (24–28)	0.06
**Postoperative complications**						
Chest re-entry, *n*. (%)	19 (23)	4 (22)	1	13 (17)	4 (8)	0.12
Permanent neurologic deficit, *n*. (%)	9 (11)	2 (11)	1	7 (9)	8 (15)	0.32
Splanchnic ischemia, *n*. (%)	2 (2)	0	1	4 (5)	3 (6)	1
Atrial fibrillation, *n*. (%)	38 (46)	7 (39)	0.61	25 (33)	23 (43)	0.25
AKI, *n*. (%)	35 (45)	11 (61)	0.21	30 (40)	31 (59)	0.04
Dialysis, *n*. (%)	15 (18)	6 (33)	0.16	11 (15)	12 (23)	0.25
Median ICU stay, days (min–max)	5 (1–34)	7 (4–20)	0.24	5 (1–129)	7 (2–57)	0.02
Median hospital stay, days (min–max)	17 (1–51)	23 (12–31)	0.27	17 (6–129)	19 (10–59)	<0.001
30-day mortality, *n*. (%)	13 (15)	3 (15)	1	6 (8)	1 (1)	0.24

AA = Ascending aorta; ET = Elephant trunk; CPB = Cardiopulmonary bypass; ACC = Aortic cross-clamp; AKI = Acute kidney injury; ICU = Intensive care unit.

**Table 4:** Characteristics of patients having a frozen elephant trunk procedure (*n* = 15).

	*p* Value
Median age, years (min–max)	55 (28–74)
Male sex, *n*. (%)	13 (87)
**Risk factors**	
Dyslipidemia, *n*. (%)	2 (13)
Obesity, *n*. (%)	6 (40)
Diabetes, *n*. (%)	1 (7)
Hypertension, *n*. (%)	13 (87)
Chronic kidney damage, *n*. (%)	1 (7)
Chronic AF, *n*. (%)	0
Smoking habitus, *n*. (%)	0
Chronic OAC, *n* (%)	0
Bicuspid aortic valve, *n*. (%)	1 (7)
Connective tissue disorder, *n*. (%)	1 (7)
Previous cardiac surgery, *n*. (%)	1 (7)
**Clinical presentation**	
Syncope, *n*. (%)	2 (13)
Transient neurological deficit, *n*. (%)	7 (47)
Coma, *n*. (%)	1 (7)
Cardiac tamponade/shock, *n*. (%)	1 (7)
Chest pain, *n*. (%)	12 (80)
AR ≥ moderate, *n*. (%)	4 (33)
Acute kidney failure, *n*. (%)	3 (20)
**Arterial cannulation**	
Right axillary, *n*. (%)	15 (100)
Femoral, *n* (%)	-
Other, *n*. (%)	-
**Cerebral perfusion**	
Retrograde	0
Selective antegrade	15 (100)
**Intraoperative data**	
Median CPB time, minutes (min–max)	195 (168–476)
Median ACC time, minutes (min–max)	125 (96–258)
Median arrest time, minutes (min–max)	29 (19–60)
Median core temperature, °C (min–max)	27 (25–29)
**Postoperative complications**	
Chest re-exploration, *n*. (%)	0
Splancnic ischemia, *n*. (%)	0
Atrial fibrillation, *n*. (%)	4 (27)
Acute kidney injury, *n*. (%)	5 (33)
Dialysis, *n*. (%)	0
Permanent neurologic deficit, *n*. (%)	1 (7)
Median ICU stay, days (min–max)	5 (2–18)
Median hospital stay, days (min–max)	20 (10–37)
30-day mortality, *n*. (%)	0

AF = Atrial fibrillation; OAC = Oral anticoagulants; AR = Aortic regurgitation; CPB = Cardiopulmonary bypass: AAC = Aortic cross-clamp; ICU = Intensive care unit.

**Table 5:** Univariable and multivariable logistic regression analysis of baseline covariates associated with 30-day mortality.

	Univariable		Multivariable	
	OR (95% CI)	*p* Value	OR (95% CI)	*p* Value
Age	1.02 (0.99–1.06)	0.23		
Male gender	1.45 (0.60–3.52)	0.41		
LVEF	0.96 (0.90–1.04)	0.37		
Chronic renal failure	1.08 (0.13–8.83)	0.94		
Previous cardiac surgery	2.27 (0.46–11.22)	0.32		
Bicuspid aortic valve	3.40 (0.85–13.58)	0.08		
Chronic AF	2.87 (0.95–8.63)	0.06		
Arterial hypertension	3.62 (1.44–9.06)	0.006		
Smoke	1.074 (0.41–2.78)	0.88		
Obesity	1.07 (0.37–3.08)	0.90		
Diabetes	3.40 (0.86–13.47)	0.08		
Dyslipidemia	0.24 (0.03–1.83)	0.17		
COPD	2.91 (0.87–9.69)	0.08		
Tamponade/shock	6.42 (2.43–17.03)	<0.001	7.30 (2.42–22.00)	<0.001
Syncope	0.39 (0.08–1.74)	0.22		
Neurological damage	1.79 (0.69–4.65)	0.23		
≥moderate aortic regurgitation	2.50 (1.00–6.23)	0.05		
Aortic arch replacement	2.34 (0.76–7.14)	0.13	11.96 (1.60–30.56)	0.02
CPB time	1.02 (1.01–1.03)	0.002		
ACC time	1.02 (1.01–1.03)	0.002	1.04 (1.02–1.06)	<0.001
Circulatory arrest	1.01 (1.00–1.02)	0.03		
Antegrade cerebral perfusion	2.51 (0.57–11.17)	0.22		
Retrograde cerebral perfusion	0.36 (0.08–1.62)	0.18		

LVEF = Left ventricular ejection fraction; AF = Atrial fibrillation; COPD = Chronic obstructive pulmonary disease; CPB = Cardiopulmonary bypass; ACC= Aortic cross-clamp.

**Table 6:** Incidence and type of redo procedures in the 2 groups.

Type	Group 1 (*n* = 163)	Group 2 (*n* = 75)	*p* Value
Proximal reintervention, *n*. (%)	4 (2)	1 (1)	0.95
Distal reintervention, *n*. (%)	9 (6)	-	-
TEVAR, *n*. (%)	5 (3)	8 (11)	0.017

TEVAR = Thoracic endovascular aortic repair.

## Data Availability

Data available on request due to restrictions privacy. The data presented in this study are available on request from the corresponding author. The data are not publicly available due to privacy.
